# Exopolysaccharide-producing strains alter heavy metal fates and bacterial communities in soil aggregates to reduce metal uptake by pakchoi

**DOI:** 10.3389/fmicb.2025.1595142

**Published:** 2025-06-26

**Authors:** Heyun Zhang, Junqing Zhang, Shuangjiao Tang, ZhongYan Deng, Randa S. Makar, Lunguang Yao, Hui Han

**Affiliations:** ^1^Henan Key Laboratory of Ecological Security for Water Source Region of Mid-line of South-to-North Diversion Project, Collaborative Innovation of Water Security for the Water Source Region of the Mid-line of the South-to-North Diversion Project of Henan Province, Nanyang Normal University, Nanyang, China; ^2^PLA, Beijing, China; ^3^Soils and Water use Department, Agricultural and Biological Research Institute, National Research Centre, Cairo, Egypt

**Keywords:** exopolysaccharide-producing strain, soil aggregates, Cd and Pb, immobilization, pakchoi

## Abstract

The characteristics of heavy metals in soil aggregates represent critical factors influencing the uptake of heavy metals by crops. However, the mechanisms underlying the immobilization of Cd and Pb by soil aggregates of different particle sizes mediated by exopolysaccharide (EPS)-producing bacteria have remained poorly understood. In this study, a selective medium was employed to isolate and screen EPS-producing bacteria from the heavy metal-contaminated soil, with their mechanisms of Cd and Pb immobilization investigated through solution adsorption experiments. Pot experiments combined with high-throughput sequencing technology were conducted to examine the effects of these strains on heavy metal uptake by pakchoi and to elucidate the underlying microbiological mechanisms. Two high-EPS-yielding bacterial strains, *Pseudomonas* sp. H7 and *Agrobacterium* sp. Z22, were successfully isolated from heavy metal-contaminated farmland. These strains effectively facilitated the formation of Fe_2_Pb(PO_4_)_2_, CdCO_3_, and Pb_2_O_3_ precipitates, thereby immobilizing Cd and Pb in aqueous solutions. Compared to the CK group, inoculation with *Pseudomonas* sp. H7 and *Agrobacterium* sp. Z22 reduced the Cd (30.7–81.8%) and Pb (8.1–57%) contents in the pakchoi tissues. Notably, *Pseudomonas* sp. H7 and *Agrobacterium* sp. Z22 enhanced EPS production and promoted the specific formation of CdCO_3_, PbCO_3_, Cd_2_(OH)_2_CO_3_, and 2PbCO_3_·Pb(OH)_2_ within microaggregates (< 250 μm), which significantly reducing Cd and Pb uptake by pakchoi. Microaggregates exhibited predominant accumulation of Cd and Pb were in organic matter-bound and residual states, whereas in macroaggregates (> 250 μm), these metals were primarily associated with Fe-Mn oxide-bound and residual states. Furthermore, inoculation with these strains altered the bacterial community composition, specifically increasing the relative abundance of Proteobacteria, *Sphingomonadaceae*, and *Micrococcales* in microaggregates, which further contributed to the reduction of Cd and Pb uptake by pakchoi. These findings provide both valuable bacterial resources and a soild theoretical foundation for developing safe vegetable production strategies in heavy metal-contaminated fields.

## 1 Introduction

The extensive use of pesticides and fertilizers in agriculture, coupled with the improper discharge of industrial wastewater, has led to severe soil heavy metal pollution (Dong et al., 2024). Among the heavy metals, cadmium (Cd) and lead (Pb) exhibit particularly persistent and pose significant threats to soil ecosystems. In China, the overall exceedance rate of heavy metal pollution in agricultural soil is as high as 16%, with Cd and Pb exceedance rates reaching 7 and 1.5%, respectively (Xia et al., 2024; Yang et al., 2023). These metals in soil can be absorbed by plants and subsequently enter the human body through multiple pathways, thereby causing substantial health risks (Sonkar et al., 2024). To address this challenge, *in situ* passivation of heavy metals has emerged as the most feasible method for remediating contaminated soils (Zhou et al., 2023). Various passivating agents, including biochar, nanomaterials, apatite, straw, and microorganisms, have been extensively investigated for their remediation efficacy (Hong et al., 2022; Wu G. et al., 2024). Notably, microbial remediation agents are gaining prominence due to their rapid reproduction, multifunctional byproducts, low cost, and environmental friendliness, which collectively enhance their applicability for immobilizing heavy metals in contaminated soils (Taharia et al., 2024; Zhao et al., 2025).

Exopolysaccharides (EPS) are high-molecular-weight substances secreted by eukaryotic or prokaryotic microorganisms during their growth and metabolic processes. These biopolymers typically form a gelatinous layer or biofilm structure surrounding the cell surface, primarily composed of polysaccharides, proteins, and nucleic acids (Wang et al., 2024b). The interaction between EPS and heavy metals constitutes a critical mechanism for microorganisms remediation of heavy metal pollution, functioning primarily through biological adsorption and biotransformation (Li et al., 2024; Wang L. et al., 2024; Yue et al., 2024). EPS contain abundant functional groups including hydroxyl and carboxyl groups, which enable binding with heavy metal cations via ion exchange, complexation, precipitation, and other interfacial reactions, thereby effectively enhancing the adsorption capacity and retention efficiency of heavy metal ions (Priyadarshanee and Das, 2024). Notably, EPS-producing strains are predominantly derived from genera such as *Rhizobium*, *Acetobacter*, *Streptococcus*, *Lactococcus*, *Lactobacillus*, *Pseudomonas*, *Bacillus*, *Sphingomonas*, and *Bifidobacterium* (Effendi et al., 2023; Mathivanan et al., 2021; Tyagi et al., 2020). Given the escalating global prevalence of heavy metal pollution in soils, the strategic application of EPS-producing bacteria represents an emerging and promising approach for remediating Cd- and Pb-contaminated soils.

The accumulation of Cd and Pb in soil not only compromises soil quality but also modifies its physicochemical properties. The distribution and stability of soil aggregates with varying particle sizes are intrinsically associated with soil quality parameters and physicochemical characteristics (Shen et al., 2022). Metal enrichment in soil aggregates directly modulates the migration dynamics of Cd and Pb, ultimately determining their environmental footprint (Wang et al., 2021). Extensive studies have established that soil aggregate size significantly affects the distribution of heavy metals, with microaggregates exhibiting a greater capacity to enrich heavy metals compared to macroaggregates (Acosta et al., 2011; Cheng et al., 2020). A notable example involves the inoculation with EPS-secreting *Pseudomonas putida* GAP-P45, which increased soil aggregate stability by over 50% (Sandhya and Ali, 2015). EPS play a crucial role in the distribution of soil macroaggregates and microaggregates, thereby influencing the enrichment of heavy metals. Nevertheless, the precise impact of EPS-producing bacteria on soil aggregate size distribution and associated heavy metal content remains insufficiently characterized and requires further investigation. Additionally, pore water, which refers to groundwater within the pores of loose sediment particles, is a vital component of soil. The concentration of heavy metals in pore water reflects the overall pollution status of soil and the migration and transformation patterns of heavy metals (Pan et al., 2021; Tang et al., 2016). EPS are closely associated with the concentration of heavy metals in pore water, yet further studies are required to determine the specific impact of EPS-producing bacteria on heavy metal content in soil pore water.

Given that the effects of EPS-producing bacteria on the particle size distribution of rhizosphere soil aggregates and the speciation of Cd and Pb remained unclear, this study pursued the following objectives: (1) to isolate high-EPS-producing bacterial strains and elucidate their mechanisms for immobilizing Cd and Pb; (2) to evaluate the dose-dependent effects of EPS-producing bacteria on Cd bioaccumulation and Pb uptake efficiency in pakchoi tissues; and (3) to examine the causal relationships between EPS-producing bacteria colonization, soil aggregate size reorganization, and heavy metal immobilization mechanisms mediated by aggregate fractions. These findings provide both valuable bacterial resources for engineering novel microbial fertilizers and critical theoretical support for optimizing EPS-producing strains in field-scale remediation of heavy metal contaminated soils.

## 2 Materials and methods

### 2.1 Screening of EPS-producing bacteria

Two grams of soil samples (moist soil, 35°03′N, 112°61′E) collected from Jiyuan city, Henan Province, were added to a sterile 50 mL shake flask and shaken to prepare a soil suspension. Soil properties: 1.37 mg kg^−1^ Cd, 97.6 mg kg^−1^ Pb, pH 7.42, 23.56 g kg^−1^ organic matter, 0.64 g kg^−1^ available P, 1.45 g kg^−1^ exchangeable Ca and 38.2 cmol(+) kg^−1^ cation-exchange capacity. A 0.1 mL aliquot of each gradient dilutions was spread onto a solid nitrogen-containing medium plate using a coating rod (Zhang H. et al., 2024). The plates were incubated at 30°C for 5 days. Colonies with distinct morphological characteristics were isolated and purified to complete the preliminary screening of EPS-producing bacteria. The bacterial culture in the logarithmic growth phase was mixed with sterilized 80% glycerol (80 mL pure glycerol + 20 mL sterile water) at a volume ratio of 1:1 and stored in a − 80°C freezer for preservation.

### 2.2 Biological characteristics of the strains

Fifty milliliters of LB medium supplemented with 5 mg L^−1^ Cd (Cd(NO_3_)_2_) and 10 mg L^−1^ Pb (Pb(NO_3_)_2_) was prepared. The growth container was 250 mL conical flask (50 mL culture solution). A bacterial suspension (OD_600_ = 1) was inoculated into the medium at a 2% ratio and incubated in a shaker at 30°C and 180 rpm for 48 h. The concentrations of Cd and Pb in the supernatant were measured via inductively coupled plasma atomic emission spectrometry (ICP–AES, ICPE-9820, Japan). The polysaccharide content in culture solution was determined using the sulfuric acid-anthrone colorimetric method (Wang et al., 2016). The strains were sequenced and identified via 16S rRNA analysis (Teng et al., 2019). Heavy metal resistance studies were conducted with Cd (50–500 mg L^−1^, in 50 mg L^−1^ increments) and Pb (1000–1800 mg L^−1^, in 100 mg L^−1^ increments) to determine the lethal concentration (LC₅₀, refers to the concentration of a chemical substance in the environment that causes the death or loss of metabolic activity in organisms). The effects of antibiotics on strain growth were also investigated (Subbaram et al., 2017). The determination of the ability of bacterial strains to secrete indole-3-acetic acid (IAA) was based on method of Jiang et al. (2008). The production of siderophores and 1-amino-1-cyclopropanecarboxylic acid (ACC) deaminase by the strains were determined according the approach proposed by Rajkumar et al. (2006) and Chretien et al. (2024).

### 2.3 Immobilization of cd and Pb by the strains

*Pseudomonas* sp. H7 and *Agrobacterium* sp. Z22 were inoculated into LB liquid medium supplemented with 5 mg L^−1^ Cd and 10 mg L^−1^ Pb for a 9-day shake flask experiment. Foue concentrations of Cd^2+^ and Pb^2+^ (10, 20, 50, and 100 mg L^−1^) were tested. Three treatment groups were established: a control group (CK), an experimental group inoculated with *Pseudomonas* sp. H7 (H7), and an experimental group inoculated with *Agrobacterium* sp. Z22 (Z22). Samples were collected on days 1, 3, 5, 7, and 9. The total heavy metal content in the culture medium (H₁) was calculated as the product of the total culture volume and the initial heavy metal concentration. For the heavy metal content in the supernatant (H₂), the culture was centrifuged, and the supernatant was collected for analysis using ICP-AES; H₂ was then determined by multiplying the supernatant’s heavy metal concentration by the total culture volume. To quantify the intracellular heavy metal content (H₃), the pelleted bacterial cells were washed three times with 5 mL of 10 mmol L^−1^ EDTA-2Na solution, freeze-dried, and weighed. The dried cells were digested with 3 mL HNO₃ and 1 mL HCl, and the heavy metal concentration in the digestate was measured via ICP-AES. The extracellular heavy metal content (H₄) was derived by subtracting H₂ and H₃ from H₁ (H₄ = H₁ − H₂ − H₃) (Han et al., 2020).

### 2.4 Mechanisms by which the strains immobilize cd and Pb in culture media

Cell pellets were collected and fixed in 15 mL of 2.5% glutaraldehyde at 30°C for 3 h. The pellets were then dehydrated using a gradient of absolute ethanol. After drying and gold coating, the samples were analyzed using scanning electron microscopy coupled with energy-dispersive X-ray spectroscopy (SEM-EDS) (Zhu et al., 2016). Changes in surface functional groups were analyzed using Fourier-transform infrared spectroscopy (FTIR) (Chakravarty and Banerjee, 2012). X-ray diffraction (XRD) analysis was performed at a scanning speed of 2° min^−1^ and a scanning angle of 5–100° (Chakravarty and Banerjee, 2012). Changes in the chemical forms of elements before and after heavy metal stress were characterized using X-ray photoelectron spectroscopy (XPS) (Zhang et al., 2022).

### 2.5 Pot experiment of pakchoi

Soil samples (moist soil, 35°03′N, 112°61′E) were collected from farmland near a factory in Jiyuan City, Henan Province. Soil properties: 1.37 mg kg^−1^ Cd, 97.6 mg kg^−1^ Pb, pH 7.42, 23.56 g kg^−1^ organic matter, 0.64 g kg^−1^ available P, 1.45 g kg^−1^ exchangeable Ca and 38.2 cmol(+) kg^−1^ cation-exchange capacity. Each pot was filled with 4 kg of soil sieved through a 2 mm mesh. Four treatment groups were established: a control group (CK), groups inoculated with strain *Pseudomonas* sp. H7 (H7) or *Agrobacterium* sp. Z22 (Z22), and a group inoculated with both strains (H7 + Z22). Pakchoi seeds were sown, and after germination, seedlings were thinned to five per pot. 40 mL bacterial suspension (OD_600_ = 1.0, 1×10^8^ CFU mL^−1^) was added to the rhizosphere soil. The control group was added with the same volume of sterile deionized water. The experiment lasted 50 days. Soil samples were collected at depths of 5–15 cm, and the heavy metal content and pH of soil pore water and leachate were measured on days 0, 15, 30, and 50. Leachate was collected from the bottom of the pots, and pore water was extracted using a Rhizon MOM soil solution sampler (AgriEco Apptec (Shanghai) LLC, China). The pH of leachate and pore water was measured using a pH meter, and heavy metal concentrations were determined using ICP-AES. After washing the mature pakchoi, the edible parts were separated from the roots. The roots were soaked in 0.01 mmol L^−1^ EDTA-2Na solution for 10 min to remove the adsorbed heavy metals on the surface. After being washed with deionized water, the whole plant was dried at 80°C to a constant weight, and the biomass of each part was measured. After crushing, 0.1 g of the sample was weighed into a polytetrafluoroethylene crucible. Mixed acid (HNO_3_-HCl-HClO-HF) was added at a ratio of 4.5:1.5:2:2. The temperature was raised for digestion until nearly dry, and the volume was made up to 5 mL. The contents of Cd and Pb were determined by ICP-AES. The vitamin C content of pakchoi was determined by 2,4-dinitrobenzhydra (DNP) method (Koo et al., 2024). The soluble protein content was determined by coomassie brilliant blue method (Masih et al., 2002).

### 2.6 Separation of soil aggregates of different particle sizes

The water stability of soil aggregates was evaluated using a wet sieving method (Cheng et al., 2023). Microaggregates (<250 μm) and macroaggregates (>250 μm) were collected using a soil aggregate analyzer, and their dry weights were measured. EPS in soil aggregates were extracted using a cation exchange resin (CER) (Vardharajula and Shaik, 2014), and polysaccharide content was determined using the sulfate-anthrone method (Wang et al., 2016). Two grams of soil aggregates were mixed with 5 mL of deionized water, and the pH of the supernatant was measured using a pH meter. Organic matter content was determined using the potassium dichromate oxidation method (Wang et al., 2024a).

### 2.7 Determination of the contents of heavy metals in the soil aggregates

Five grams of soil aggregates were mixed with 25 mL of extraction solution (1.967 g diethylenetriamine pentaacetic acid, 13.3 mL triethanolamine (TEA), 1.11 g anhydrous calcium chloride, and 950 mL water, pH 7.3). The supernatant was digested with 3 mL nitric acid and 1 mL hydrochloric acid and determined Cd and Pb concentrations by ICP-AES. Tessier’s sequential extraction method was used to determine the contents of exchangeable Cd/Pb (EX-Cd/Pb), carbonate-bound Cd/Pb (CB-Cd/Pb), Fe-Mn oxide-bound Cd/Pb (Fe-Mn-Cd/Pb), organic matter-bound Cd/Pb (OMB-Cd/Pb), and residual Cd/Pb (RES-Cd/Pb) in rhizosphere soil (Tessier et al., 1979). The Cd and Pb contents in these extractions was also determined by ICP-AES.

### 2.8 Electron microscopy characterization of soil aggregates

The morphology of soil aggregates was analyzed using a JSM-7900F scanning electron microscope. The interaction between metal ions and EPS was investigated using an Aqualog fluorescence spectrophotometer to measure the 3D-EEM spectra of EPS (Peng et al., 2016).

### 2.9 Determination of bacterial community in soil aggregates

Bacterial community analysis was performed on soil from fresh large aggregates (control group: CK-B; experimental groups: H7-B, Z22-B, H7 + Z22-B) and microaggregates (control group: CK-S; experimental groups: H7-S, Z22-S, H7 + Z22-S). Microbial DNA was extracted from soil aggregates using the E. Z. N. A.® soil DNA Kit (Omega Bio-tek, Norcross, GA, United States) according to manufacturer’s protocols. The V3-V4 region of the 16S rRNA gene was amplified using primers 338F (5′- ACTCCTACGGGAGGCAGCAG-3′) and 806R (5’-GGACTACHVGGGTWTCTAAT-3′). All samples were mixed with PCR products, and then subjected to electrophoresis on a 2% agarose gel. The gel was cut using AxyPrepDNA Gel Recovery Kit (AXYGEN Company) to recover the PCR products. These products were then quantified using the QuantiFluor™ -ST Blue Fluorescent Quantitative System (Promega Company). Subsequently, samples were mixed in proportion based on their sequencing requirements, followed by library construction, and finally sequenced at higher levels (Liang et al., 2022). Sequencing results were analyzed on the Meiji Biotech website.^1^

### 2.10 Data analysis

Data were analyzed using Excel 2019 and SPSS 26.0. Mathematically processed results are presented in the form of M ± SE. Before performing Tukey’s multiple comparison test, Levene’s test was applied to assess the homogeneity of variances across treatment groups (significance level *α* = 0.05). Origin 2024 and Excel software were used for image processing. Advantage software was used for XPS data analysis and Matlab 2019a software was used for 3D fluorescence spectroscopy analysis. PCA analysis (Principal Component Analysis, R language 3.3.1) was used for the differences among samples of multiple sets of data. UPGMA (Unweighted Pairing-Group Method with Arithmetic Mean, Qiime 2020.2.0) is a clustering analysis method used to solve classification problems. LEfSe^2^ is based on the taxonomy of the samples according to the different conditions of grouped linear discriminant analysis (LDA).

## 3 Results

### 3.1 Isolation and identification of polysaccharide-producing bacteria

**Figure 1:**
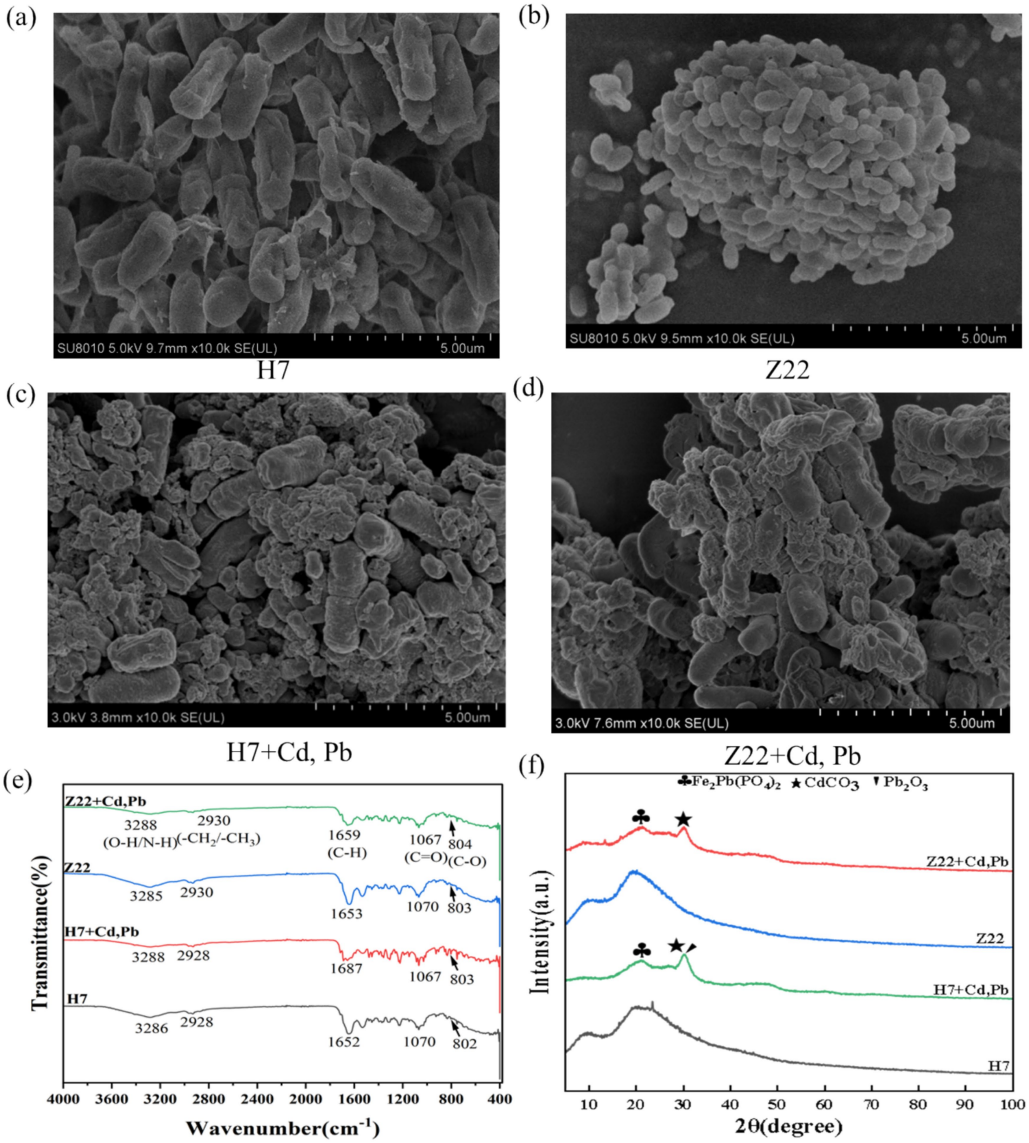
Mechanisms of immobilization of Cd and Pb by strains H7 and Z22. **(a)** SEM image of strain H7; **(b)** SEM image of strain Z22; **(c)** SEM image of strain Z22 + Cd, Pb; **(d)** SEM image of strain Z22 + Cd, Pb; **(e)** FTIR images of strains H7 and Z22; **(f)** XRD images of strains H7 and Z22.

Eight bacterial strains were selected based on their ability to adsorb heavy metals and produce EPS. The Cd removal rates of these strains ranged from 64.87 to 86.29%, while the Pb removal rates ranged from 56.66 to 86.84% in solutions containing 5 mg L^−1^ Cd and 10 mg L^−1^ Pb (Supplementary Figure S1). The EPS production of these strains varied between 147.63 and 267.48 mg L^−1^, with strains H7 and Z22 exhibiting EPS contents of 183.71 mg L^−1^ and 267.48 mg L^−1^, respectively (Figure 1). Consequently, strains H7 and Z22 were chosen as the target strains for further investigation. Based on phylogenetic analysis, strain H7 was identified as *Pseudomonas* sp. (PP784325), while strain Z22 was identified as *Agrobacterium* sp. (PP784326) (Supplementary Figure S2). The lethal concentrations (LC_50_) of Cd and Pb for strain H7 were determined to be 400 mg L^−1^ and 1700 mg L^−1^, respectively, whereas the LC values for strain Z22 were 300 mg L^−1^ and 1,600 mg L^−1^, respectively (Supplementary Table S1).

### 3.2 Adsorption of heavy metals by polysaccharide-producing bacteria

By the seventh day of the experiment, strains H7 and Z22 significantly (*p* < 0.05) reduced the Cd concentrations in the solution by 68.2 and 53.6%, respectively, compared to the control (CK) group (Supplementary Figure S3a). Similarly, the Pb concentrations were significantly (*p* < 0.05) reduced by 74.6 and 49.8%, respectively (Supplementary Figure S3b). At low heavy metal concentrations (10 mg L^−1^ and 20 mg L^−1^), both strains primarily reduced heavy metals through intracellular enrichment (Supplementary Figures S3c,d). However, when exposed to higher concentrations of heavy metals (50 mg L^−1^ and 100 mg L^−1^), extracellular adsorption by strains H7 and Z22 became more prominent than intracellular enrichment (Supplementary Figures S3e,f).

### 3.3 Immobilization of cd and Pb by polysaccharide-producing bacteria in solution

Under conditions without heavy metal stress, the surfaces of strains H7 and Z22 appeared smooth (Figures 1a,b). However, in the presence of Cd and Pb, their surfaces became rough, with visible precipitates forming (Figures 1c,d). FTIR analysis revealed shifts in the peaks near 3,288 cm^−1^ (O-H, N-H), 1,659 cm^−1^ (C-H), and 1,067 cm^−1^ (C=O) in the cell walls of strains H7 and Z22 after Cd and Pb adsorption, compared to cells without heavy metal exposure (Figure 1e). Specifically, the absorption peaks of C-H and C=O groups in strains H7 and Z22 shifted by 35 cm^−1^ and 6 cm^−1^, respectively, with strain H7 exhibiting more pronounced shifts than strain Z22 (Figure 1e). These results suggest that O-H, N-H, C-H, and C=O groups were involved in the immobilization of Cd and Pb. Furthermore, XRD analysis detected the presence of Fe₂Pb(PO₄)₂, CdCO₃, and Pb₂O₃ on the cell walls of strains H7 and Z22 under Cd and Pb stress (Figure 1f). Additionally, XPS analysis identified new peaks for Cd3d₃/₂, Cd3d₅/₂ (CdS), and metallic Cd in the Cd3d spectrum, as well as peaks for Pb4f₅/₂ and Pb4f₇/₂ (Pb₃O₄ and 2PbCO₃·Pb(OH)₂) in the Pb4f spectrum (Supplementary Figure S4). These findings indicate that strains H7 and Z22 facilitated the formation of precipitates such as CdS and Pb₃O₄.

### 3.4 Effects of strains on the growth and cd and Pb contents of pakchoi

**Figure 2:**
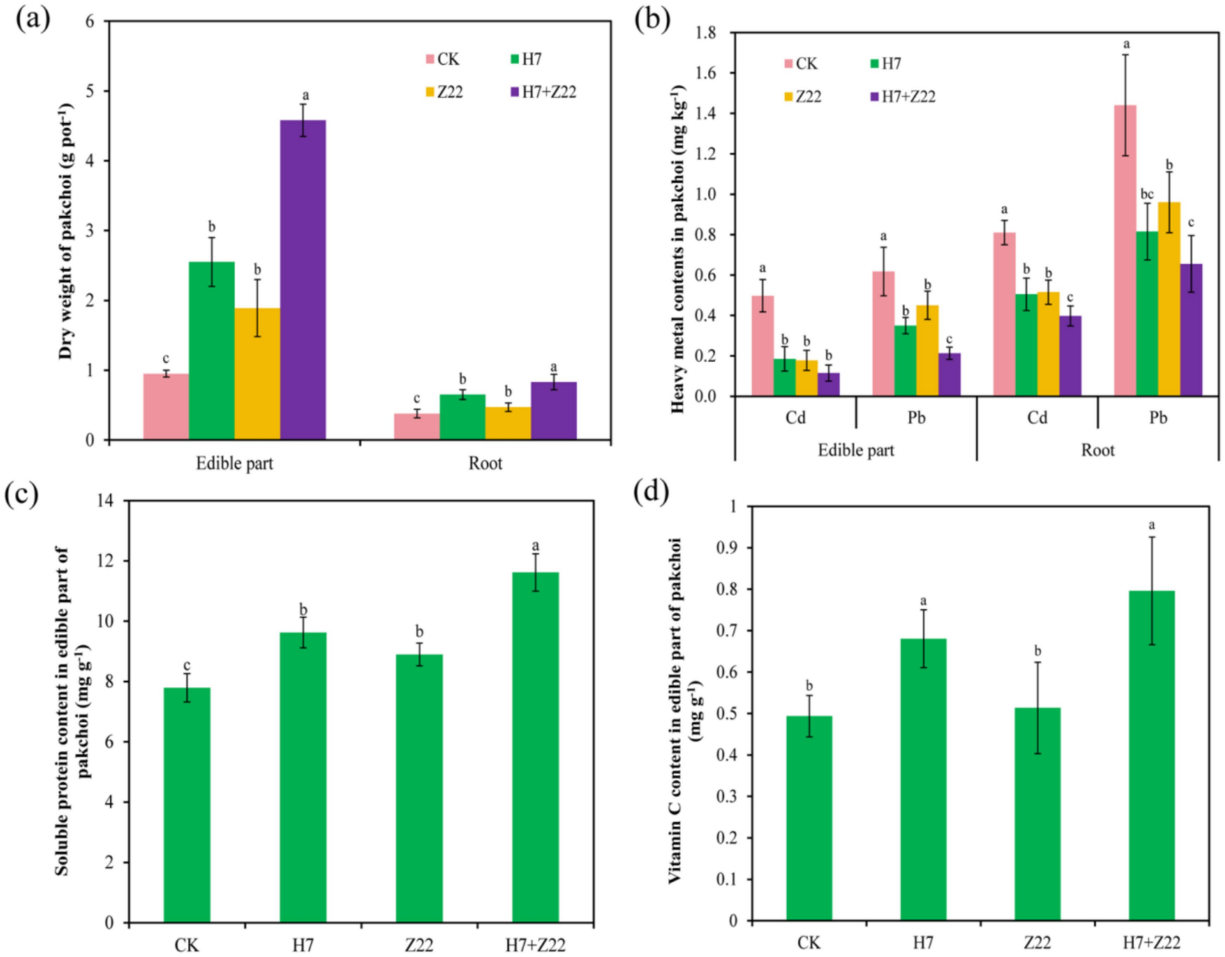
EffectS of polysaccharide-producing bacteria on the growth and Cd and Pb content of pakchoi. **(a)**: Dry weight of pakchoi; **(b)**: Cd and Pb contents in pakchoi; **(c)**: Soluble protein content in pakchoi; **(d)**: Vitamin C content of pakchoi. The values are the mean and standard deviation (*n* = 3), and one-way analysis of variance is used. Different lowercase letters indicate statistically significant differences (*p* < 0.05).

Compared to the CK group, the inoculation with H7, Z22, and H7 + Z22 significantly (*p* < 0.05) increased the dry weight of the edible parts (56.1–81.8%) and roots (8.1–55.4%) of pakchoi (Figure 2a). Inoculation also led to a reduction in Cd (30.7–68%) and Pb (31.1–57%) content in the edible parts, as well as Cd (27.9–47.2%) and Pb (39.8–57.7%) content in the roots of pakchoi (Figure 2b). In the absence of inoculation, the soluble protein content in the edible parts of pakchoi was 7.63 mg g^−1^. After inoculation with H7, Z22, and H7 + Z22, the soluble protein content significantly (*p* < 0.05) increased by 22.4, 12.6, and 32%, respectively (Figure 2c), while the vitamin C content increased by 29.6, 12.5, and 38.2%, respectively (Figure 2d). These results demonstrate that inoculation with polysaccharide-producing bacteria not only enhanced the growth of pakchoi but also improved its nutritional quality.

### 3.5 Heavy metal content and pH in soil pore water and leachate

**Figure 3:**
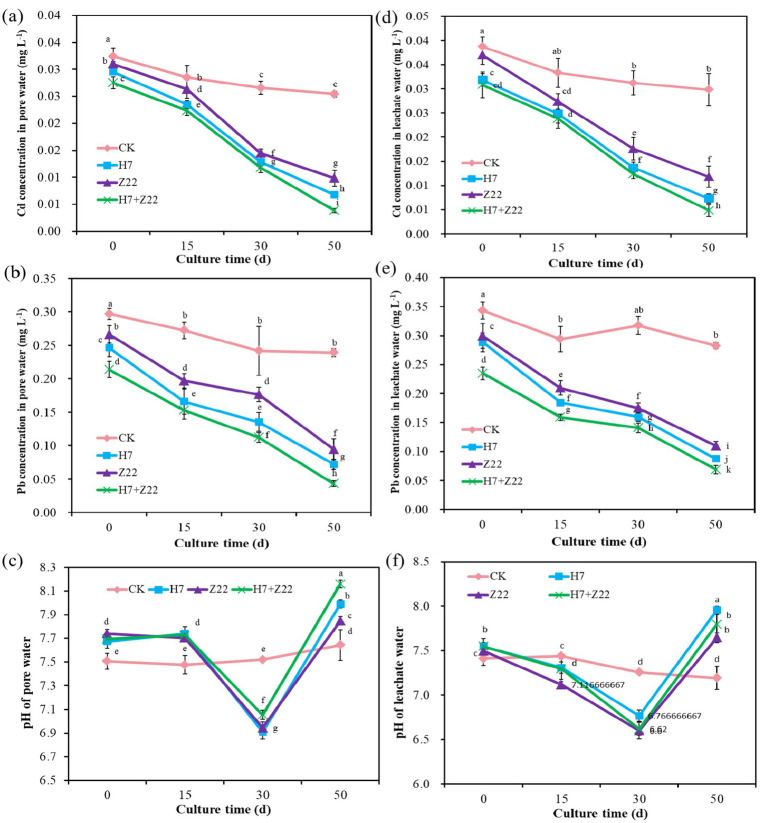
ffects of polysaccharides-producing bacteria on heavy metal content and pH in soil pore water and leached water. **(a)** Cd concentration in pore water; **(b)** Pb concentration in pore water; **(c)** pH of pore water; **(d)** Cd concentration in leachate water; **(b)** Pb concentration in leachate water; **(c)** pH of leachate water. The values are presented as the means and standard deviations (*n* = 3). Different lowercase letters indicate statistically significant differences (*p* < 0.05).

As the cultivation period progressed, the Cd and Pb content in the pore water of the H7, Z22, and H7 + Z22 treatment groups were significantly lower than those in the CK group (Figures 3a,b), indicating that strains H7 and Z22 effectively immobilized heavy metals and reduced their bioavailability. The pH of the pore water in the control group remained stable, whereas in the H7, Z22, and H7 + Z22 groups, it initially decreased from 7.68 to 7.02 and then increased to 8.12 (Figure 3c). Similarly, the Cd and Pb content in the soil leachate of the treatment groups were significantly lower than those in the CK group over time (Figures 3d,e). The pH of the soil leachate in the CK group showed no significant change, while in the treatment groups, it decreased from 7.54 to 6.76 and then increased to 7.79 (Figure 3f). These findings suggest that polysaccharide-producing bacteria influenced the pH dynamics of the soil leachate, thereby enhancing heavy metal immobilization in the soil.

### 3.6 Particle size distribution and polysaccharide content of soil aggregates

Inoculation with strains H7 and Z22 significantly (*p* < 0.05) increased the proportion of macroaggregates while reducing the proportion of microaggregates compared to the control (Supplementary Figure S5a). In the H7 + Z22 group, the polysaccharide content in macroaggregates increased from 2.7 mg kg^−1^ to 9.8 mg kg^−1^, and in microaggregates, it increased from 2.8 mg kg^−1^ to 12.3 mg kg^−1^ (Supplementary Figure S5b). Additionally, inoculation with strains H7 and Z22 significantly increased the pH of soil aggregates across different particle sizes but had no significant effect on organic matter content (Supplementary Figures S5c,d). Overall, these results indicate that inoculation with strains H7 and Z22 enhanced the polysaccharide content and heavy metal retention capacity of soil aggregates.

### 3.7 Different forms of cd and Pb in soil aggregates

**Figure 4:**
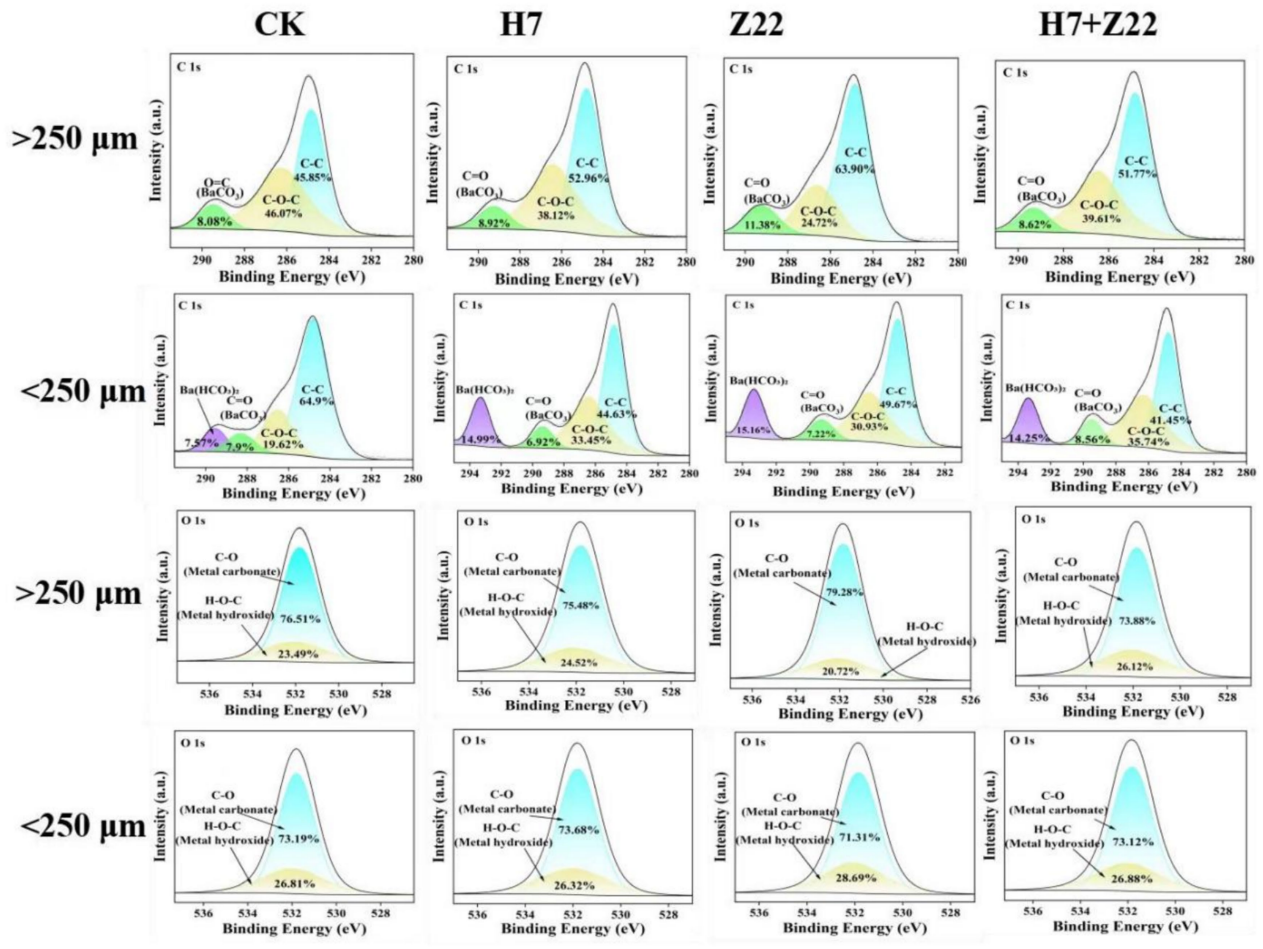
Analysis of C 1 s and O 1 s spectra for macroaggregates and microaggregates.

**Figure 5:**
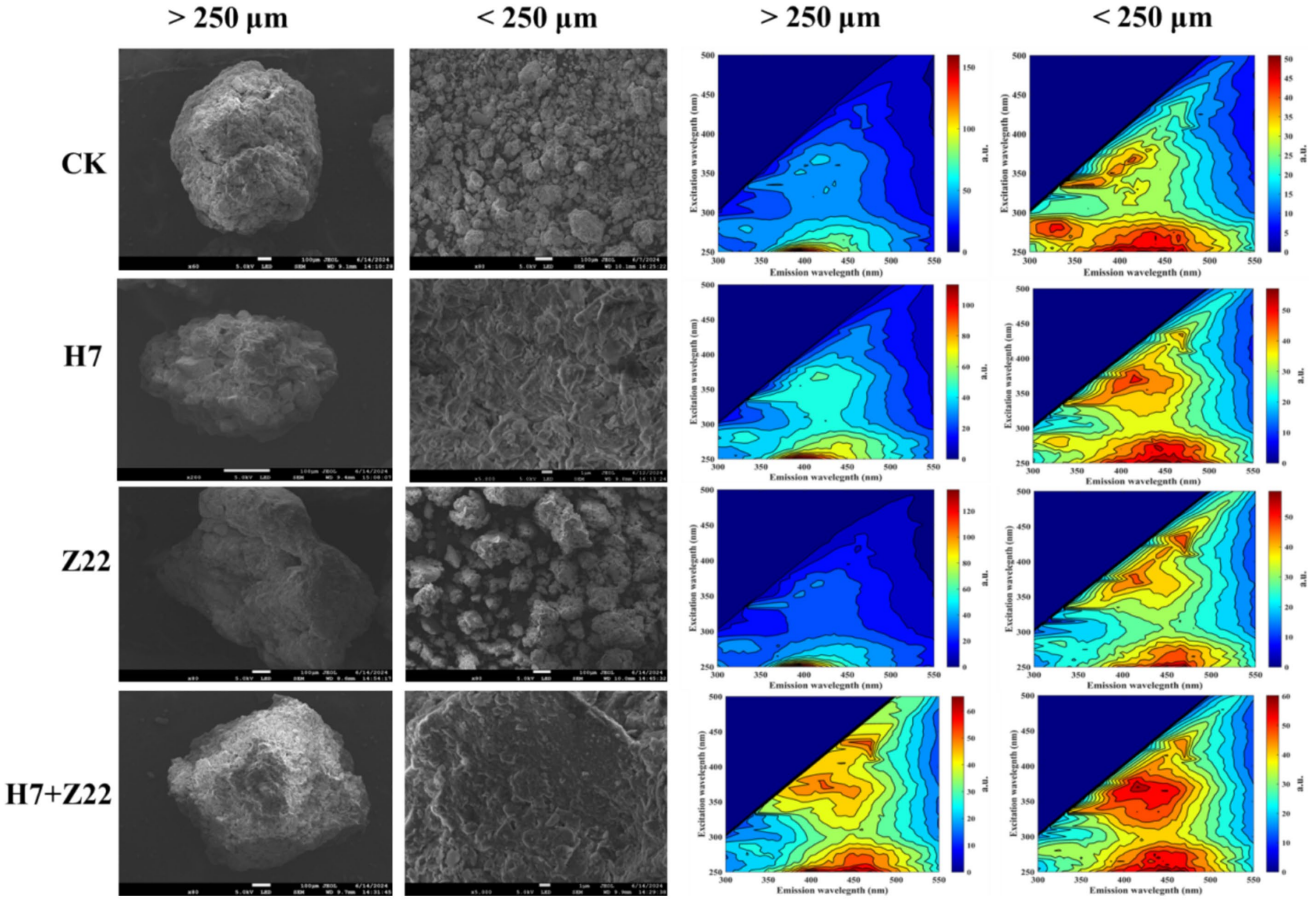
Scanning electron microscope and 3D-EEM images of soil aggregates with macroaggregates and microaggregates.

In the CK group, the DTPA-extractable Cd content in macroaggregates was 0.047 mg kg^−1^, while in microaggregates, it was 0.044 mg kg^−1^. Inoculation with strains H7 and Z22 significantly reduced the DTPA-Cd content in both macro- and microaggregates (Supplementary Figure S6a). Similarly, the DTPA-Pb content in soil aggregates was also reduced following inoculation with strains H7 and Z22 (Supplementary Figure S6b), indicating that microaggregates exhibit a greater capacity for immobilizing Cd and Pb. Over time, the EX-Cd content decreased significantly in the H7- and Z22-inoculated groups, while the Fe-Mn-Cd and RS-Cd contents increased. In the H7 + Z22 treatment, the proportion of Fe-Mn-Cd increased from 18.3 to 29.4%, and the proportion of RS-Cd increased from 21.3 to 29.1% (Supplementary Figure S6c). These results suggest that strains H7 and Z22 facilitated the transformation of bioavailable Cd in macroaggregates into Fe-Mn oxide-bound and residual forms. Additionally, in microaggregates, the proportion of OM-Cd increased from 18.6 to 27.1%, and the proportion of RS-Cd increased from 23.3 to 31.3% in the H7 + Z22 treatment (Supplementary Figure S6c), indicating that strains H7 and Z22 promoted the conversion of bioavailable Cd into organic matter-bound and residual forms. Similar trends were observed for Pb, with strains H7 and Z22 inducing the transformation of bioavailable Pb in macroaggregates into Fe-Mn oxide-bound and residual forms, and in microaggregates into organic matter-bound and residual forms (Supplementary Figure S6d). In microaggregates, the percentage of C-C fitting peaks decreased, while the percentages of C-O-C, C=O, and HCO₃^−^ fitting peaks increased. Additionally, precipitates such as CdCO₃, PbCO₃, Cd₂(OH)₂CO₃, and 2PbCO₃•Pb(OH)₂ were detected, indicating that C-O-C, C=O, and HCO₃^−^ groups were involved in the immobilization of heavy metals in microaggregates (Figure 4; Supplementary Figure S7). Compared to the control, soil macroaggregates inoculated with H7 and Z22 exhibited denser particle aggregates with smoother surfaces, while microaggregates became looser with rougher surfaces and larger specific surface areas, providing more adsorption sites. This suggests that microaggregates have a stronger capacity for adsorbing Cd and Pb (Figure 5). Previous studies have demonstrated that three-dimensional fluorescence intensity is closely associated with EPS content (Ma et al., 2018; Rigby and Smith, 2020). The fluorescence intensity of microaggregates in the H7 and Z22 treatments was significantly higher than that of macroaggregates, indicating a greater EPS content in microaggregates (Figure 5).

### 3.8 Bacterial community diversity in rhizosphere soil aggregates

**Figure 6:**
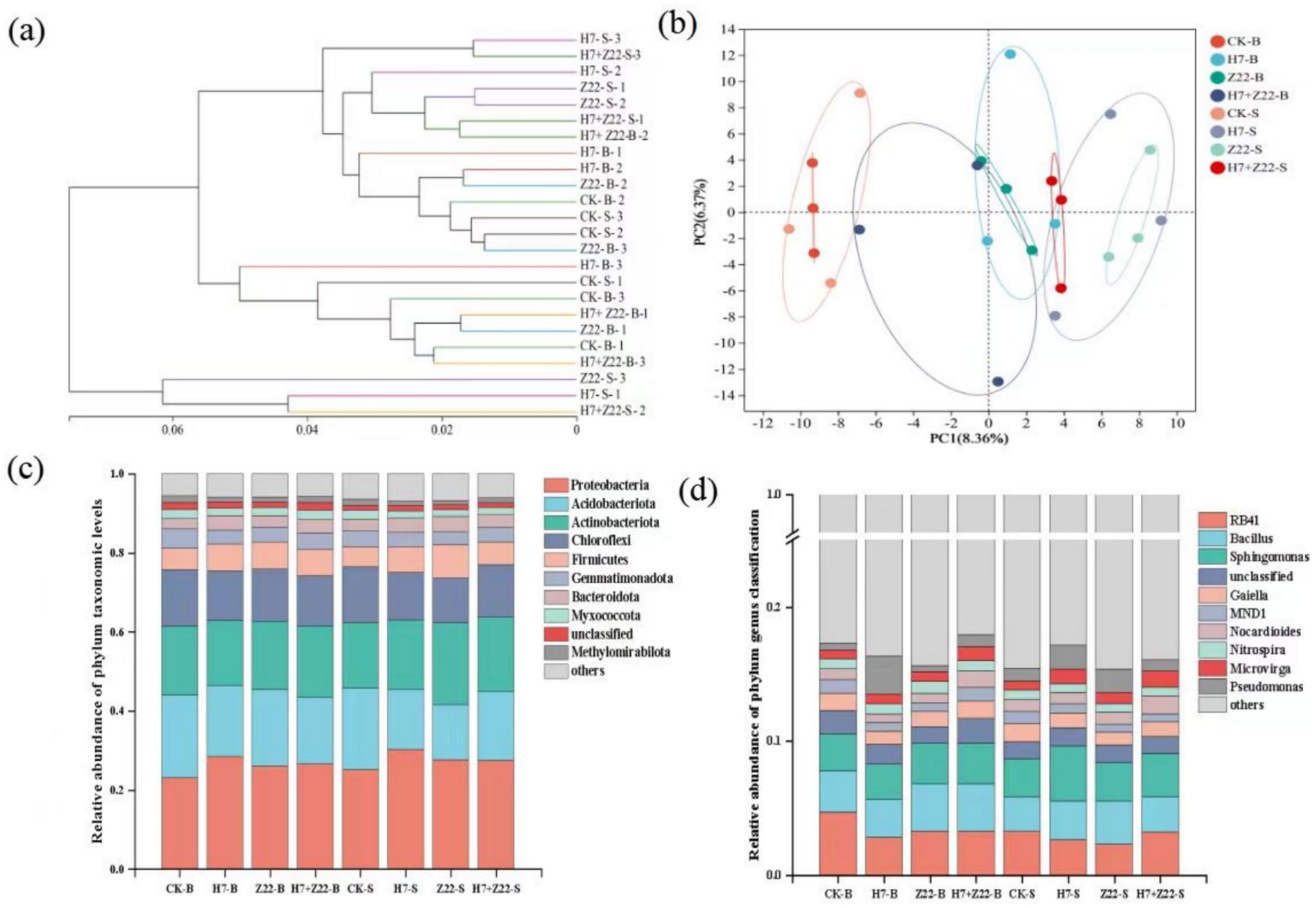
Analysis of bacterial community composition in rhizosphere soil aggregates. **(a)** UPGMA algorithm constructs tree analysis; **(b)** PCA analysis; **(c)** The relative abundance of phylum level sequences in bacterial communities; **(d)** The relative abundance of genus level sequences in bacterial communities.

**Figure 7:**
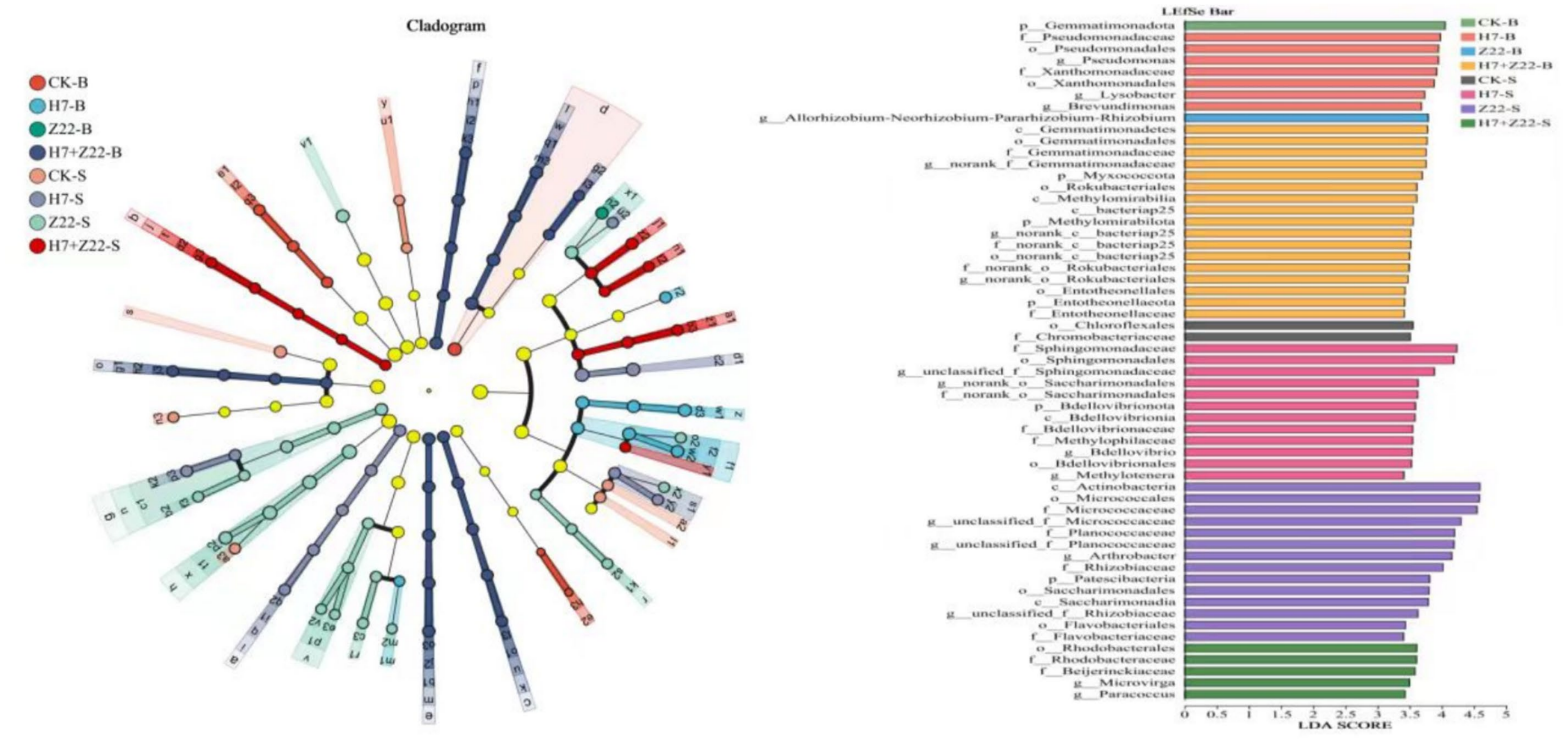
Analysis of LEfse multi-level species hierarchical tree and LDA discriminant bar chart for eight processing groups. Nodes of different colors represent microbial groups that are significantly enriched in the corresponding groups and have a significant impact on the differences between groups. The pale yellow nodes represent microbial groups that show no significant differences in different groups or have no significant effect on differences between groups. The bar chart shows the LDA values of different differentially identified species, visually presenting the extent of the influence of the characteristic species identified among different groups on the differential effect.

The UPGMA algorithm revealed that in macroaggregates, the CK group and the H7 and Z22 inoculation groups clustered on the same branch, whereas in microaggregates, they formed distinct clusters (Figure 6a). Principal component analysis (PCA) further supported these findings, showing that the H7 and Z22 inoculation groups were closer to the CK group in macroaggregates but more distant in microaggregates (Figure 6b). Inoculation with strains H7 and Z22 increased the relative abundance of Proteobacteria, Acidobacteriota, and Actinobacterota in microaggregates while reducing the relative abundance of Chloroflexi and Myxococcota (Figure 6c). At the genus level, the dominant taxa included RB41, *Bacillus*, *Sphingomonas*, *Gaiella*, *MND1*, and *Nocardioides*. Following treatment with H7 and Z22, the relative abundance of *Sphingomonas*, *Gaiella*, and *Nocardioides* in microaggregates increased significantly (Figure 6d). In microaggregates, the key bacterial groups in the H7 + Z22 treatment included *f_Planococcaceae*,*g_Arthrobacter*, *o_Rhodobacterales*, *f_Beijerinckiaceae*, *g_Microvirga*, and *g_Paracoccus* (Figure 7). Compared to macroaggregates, microaggregates exhibited a greater number of significantly different bacterial populations after inoculation with H7 and Z22, indicating a more pronounced impact of these strains on bacterial community composition in microaggregates. Previous studies have reported that *Sphingomonas* possesses the ability to degrade various heavy metals and promote plant growth (Bin, 2011; Krishnan et al., 2016; Tangaromsuk et al., 2002). Additionally, *Saccharimonadales* abundance has been linked to polysaccharide content and exhibits synergistic effects with nitrogen cycling-related genes (Wang et al., 2022).

## 4 Discussion

In this study, two bacterial strains exhibiting high EPS production capacity and demonstrating Cd and Pb immobilization ability, *Pseudomonas* sp. H7 and *Agrobacterium* sp. Z22, were isolated from the heavy metal-contaminated farmland. Through integrated solution adsorption assays and pot experiments, we demonstrated that strains H7 and Z22 effectively immobilized Cd and Pb while significantly inhibiting their uptake by pakchoi through three synergistic mechanisms: (1) cell wall adsorption, (2) EPS-mediated chelation, and (3) modulation of soil aggregate structure combined with bacterial community reconfiguration. Microbial immobilization and remediation technologies for heavy metals hold significant promise in addressing soil heavy metal pollution (Jiang et al., 2022). The primary mechanisms underlying microbial heavy metal immobilization encompass: (1) secretion of biofilms, polysaccharides, and other substances to chelate heavy metals (Zeng et al., 2020); (2) induction of heavy metal phosphate and carbonate precipitation, reducing their mobility (Huang H. et al., 2024); (3) cell wall adsorption and intracellular enrichment of heavy metals (Zhang Y. et al., 2024); and (4) redox reactions (Tan et al., 2020). In this study, strains H7 and Z22 induced the formation of Fe₂Pb(PO₄)₂, CdCO₃, and Pb₂O₃ precipitates to immobilize Cd and Pb. Additionally, these strains increased the EPS content in the soil and enhanced the capacity of microaggregates to immobilize Cd and Pb. Mohanraj et al. (2021) isolated an EPS-producing endophytic actinomycete, *Actinobacterial* sp., from heavy metal-contaminated soil, which effectively reduced total Cd and Pb levels. Similarly, the EPS-producing strain *Pseudoalteromonas* sp. decreased Pb content in the edible parts and roots of Chinese cabbage grown in Pb-contaminated soil (Cao et al., 2023). The novel findings of this study are as follows: (1) Strains H7 and Z22 immobilized Cd and Pb through cell wall adsorption, EPS secretion for chelation, and the induction of Fe₂Pb(PO₄)₂, CdCO₃, and Pb₂O₃ precipitate formation. (2) Strains H7 and Z22 increased EPS content in microaggregates, enhancing their capacity to immobilize Cd and Pb while reducing Cd and Pb uptake in pakchoi.

In this study, the EPS content of strains H7 and Z22 under Cd and Pb stress was 183.71 mg L^−1^ and 267.48 mg L^−1^, respectively, and they reduced Cd (53.6–68.2%) and Pb (49.8–74.6%) concentrations in solution. The significant reduction in Cd/Pb mobility and subsequent uptake by pakchoi mediated by strains H7 and Z22 can be attributed to the metal-binding properties of their secreted EPS. As a critical component of bacterial biofilms, EPS contains abundant functional groups (e.g., carboxyl, hydroxyl, and phosphoryl groups) that exhibit high affinity for divalent metal ions through ion exchange, surface complexation, and electrostatic interactions (Li Y. et al., 2020). Xia et al. (2020) reported that EPS possess large surface areas and numerous negatively charged functional groups, indicating their excellent capacity to adsorb Hg^2+^. In this study, XRD analysis revealed that strains H7 and Z22 generated Fe₂Pb(PO₄)₂, CdCO₃, and Pb₂O₃ precipitates under Cd and Pb stress. These findings suggest that negatively charged groups in EPS undergo ion exchange reactions with heavy metal cations and form insoluble compounds, thereby immobilizing Cd and Pb (Li Q. et al., 2020; Wang et al., 2020). Notably, the dual function of EPS—direct chemical fixation and indirect physical encapsulation—provides a more robust strategy for rhizosphere metal sequestration compared to single-mechanism bioagents.

After soils were inoculated with strains H7 and Z22, the EPSs alkalized the soil pore water and reduced the concentrations of Cd and Pb in the pore water. The observed reduction in Cd and Pb concentrations within rhizosphere leachate and pore water, coupled with elevated pH upon inoculation with strains H7 and Z22, suggested a pH-mediated geochemical regulation mechanism underpinning their metal immobilization efficacy (Si et al., 2024). EPS-producing bacteria often secrete alkaline metabolites (e.g., ammonia, carbonate ions) during EPS synthesis and nitrogen metabolism, which could neutralize soil acidity and shift metal speciation toward less mobile forms (Cai et al., 2024). The dual effect of pH elevation and EPS secretion creates a self-reinforcing immobilization loop. Our data further reveal that pH modulation synergistically amplified the adsorption capacity of EPS: higher pH increases deprotonation of carboxyl and phosphoryl groups in EPS, strengthening their electrostatic attraction to cationic Cd and Pb (Zhang et al., 2015). Compared to conventional pH-amending agents (e.g., lime), microbial pH regulation offers spatial–temporal precision by targeting root-proximal zones without inducing excessive alkalinity that harms soil microbiota (Wu et al., 2025). Nevertheless, the sustainability of this pH shift under field conditions—where rainfall leaching and organic acid exudation may counteract alkalization—requires verification.

In this study, inoculation with strains H7 and Z22 increased EPS content in soil aggregates, particularly in microaggregates (<250 μm). 3D-EEM results further confirmed that EPS content in microaggregates exceeded than in macroaggregates. The superior heavy metal immobilization capacity of microaggregates stems from their unique physical structure, chemical composition, and biological attributes (Huang X. et al., 2024). Compared to macroaggregates, microaggregates have larger specific surface areas, higher organic matter content, greater concentrations of clay minerals and iron-manganese oxides, more active microbial communities, and more stable structures (Hu et al., 2023; Huang X. et al., 2024; Wu B. et al., 2024). These synergistic properties collectively establish microaggregates as key players in soil heavy metal remediation (Wen et al., 2022). The primary mechanisms of heavy metal adsorption by soil aggregates include: (1) reaction of heavy metal ions with anions in soil aggregates to form precipitates (Zhou et al., 2020); (2) interaction of heavy metal ions with functional groups (e.g., carboxyl, hydroxyl) in organic compounds to form complexes; and (3) redox reactions between metal oxides in soil aggregates and heavy metal cations, leading to precipitation. In this study, inoculation with strains H7 and Z22 increased the number of carboxyl and hydroxyl functional groups in microaggregates, facilitating the formation of complexes with heavy metal ions. Additionally, more Cd(OH)₂, CdOHCl, Pb₂O₃, and Pb(OH)₂ precipitates were observed in microaggregates. Strains H7 and Z22 also induced the transformation of bioavailable heavy metals into Fe-Mn oxide-bound and residual forms in macroaggregates, likely due to the enrichment of iron and manganese oxides in these aggregates. Soil aggregates provide a spatially heterogeneous microenvironment for microorganisms and their activities (Lv et al., 2023). The composition and structure of microbial communities vary across aggregates of different sizes. In this study, inoculation with strains H7 and Z22 increased the relative abundance of Proteobacteria, Acidobacteriota, and Actinobacterota in microaggregates. Among these, Proteobacteria not only exhibit strong habitat adaptability but also have the potential to improve soils contaminated with heavy metals (Emenike et al., 2023; Gong et al., 2023). Li et al. (2022) found that the abundance of Sphingomonas was positively correlated with Cd, Pb, and As concentrations in soil. The high linear discriminant analysis (LDA) values of Sphingomonas in microaggregates suggest that polysaccharide-producing bacteria increased its abundance to reduce Cd and Pb levels in microaggregates. Thus, strains H7 and Z22 play a crucial role in the remediation of heavy metals in soil aggregates, reducing heavy metal uptake by vegetables and minimizing their impact on human health.

## 5 Conclusion

Two EPS-producing bacteria, *Pseudomonas* sp. H7 and *Agrobacterium* sp. Z22, were isolated from heavy metal-contaminated soil and had the ability to immobilize Cd and Pb. These strains reduced rhizosphere bioavailable Cd and Pb through direct adsorption, enhanced microaggregate formation, and reshaped bacterial community structure, collectively lowering heavy metal uptake in pakchoi, thereby offering novel microbial candidates for bioremediating contaminated farmland. This provided new candidate strain resources for microbial remediation of heavy metal-contaminated farmland. Meanwhile, by blocking the migration of heavy metals to edible parts, it has direct application value in ensuring the safety of leafy vegetable agricultural products. The EPS secreted by H7 and Z22 improved soil aggregate stability, enhancing water/nutrient retention, while restructured rhizobacterial communities favored colonization by plant growth-promoting rhizobacteria, suggesting synergistic remediation-agricultural improvement potential. However, the current research is a pot experiment. Factors such as soil heterogeneity, climate fluctuations, and competition from indigenous microorganisms in the field environment may affect the actual remediation efficacy of the strains, which requires further verification. Moreover, the causal link between EPS-mediated metal immobilization and microbial community dynamics remains unresolved, demanding integrated metagenomic/metabolomic analyses to decipher functional genes and metabolic pathways.

## Data Availability

The original contributions presented in the study are included in the article/Supplementary material, further inquiries can be directed to the corresponding author. The bacterial sequencing data were uploaded to the Sequence Read Archive (SRA) of NCBI (http://www.ncbi.nlm.nih.gov/sra) and can be accessed under the accession number PRJNA876528.

## Footnotes

^1^ http://www.majorbio.com

^2^ http://galaxy.biobakery.org/
