# Supplementary material for: Exopolysaccharide-producing strains alter heavy metal fates and bacterial communities in soil aggregates to reduce metal uptake by pakchoi
Source: Front Microbiol. 2025 Jun 26;16:1595142. doi: 10.3389/fmicb.2025.1595142 (PMC12241154; doi:10.3389/fmicb.2025.1595142)
Supplement: Supplementary file 1 [file Supplementary_file_1.docx]

Supplementary Material

Exopolysaccharide-producing strains alter heavy metal fates and bacterial communities in soil aggregates to reduce metal uptake by pakchoi

Heyun Zhang ^1^, Junqing Zhang ^1^, Shuangjiao Tang ^1^, ZhongYan Deng ^2^, Randa S. Makar ^3^, Lunguang Yao ^1^, Hui Han ^1^*

^1^Henan Key Laboratory of Ecological Security for Water Source Region of Mid-line of South-to-North Diversion Project, Collaborative Innovation of Water Security for the Water Source Region of the Mid-line of the South-to-North Diversion Project of Henan Province, Nanyang Normal University, Nanyang 473061, Henan, China

^2^PLA, Beijing 100043, China

^3^Soils and Water Use Department, Agricultural and Biological Research Institute, National Research Centre, Dokki, Cairo 12622, Egypt.

*** Correspondence:**Hui Han
hanhui2018@nynu.edu.cn

**Table S1.** Promoting characteristics and heavy metal resistance of functional strains

|  | H7 | Z22 |
| --- | --- | --- |
| IAA (mg L^-1^) | 67.5 | 56.7 |
| Siderophore | ++++ | +++++ |
| ACC deaminase | + | + |
| Lethal concentration of Cd (mg L^-1^) | 400 | 300 |
| Lethal concentration of Pb (mg L^-1^) | 1700 | 1600 |
| Gram stain | - | + |
| Kanamycin | + | - |
| Tetracycline | + | + |
| Chloramphenicol | + | + |

IAA: indole-3-acetic acid; ACC deaminase: 1-amino-1-cyclopropanecarboxylic acid deaminase; “+” indicating the production, positivity, or resistance, “-” indicating no production, negative, or lack of resistance.

**F****IGURE S1** Removal rate of heavy metals and polysaccharide content of 8 test strains. The values are presented as the means and standard deviations (n = 3) and the data followed by different lowercase letters in same indicator were significantly different (*P* < 0.05) according to Tukey’s test.


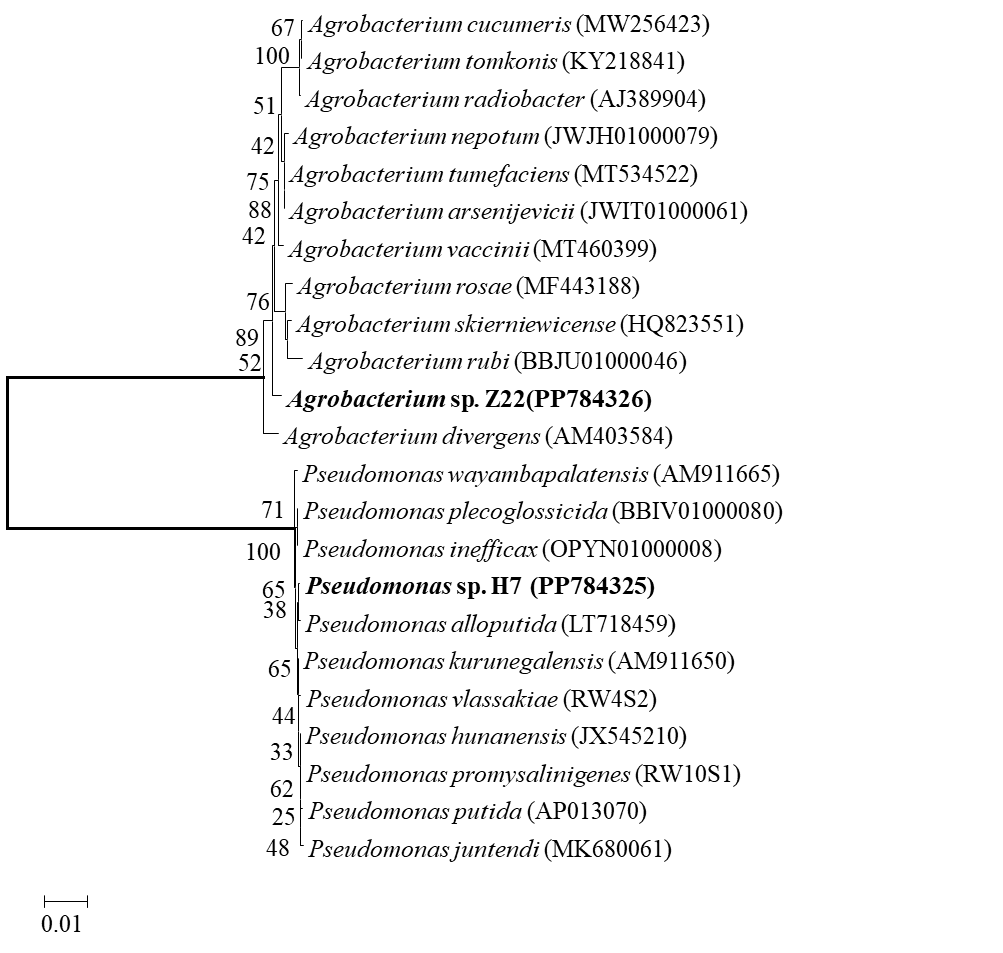


**FIGURE S2** Phylogenetic tree constructed by the Neighbor Joining method on the basis of 16S rDNA sequences of strains


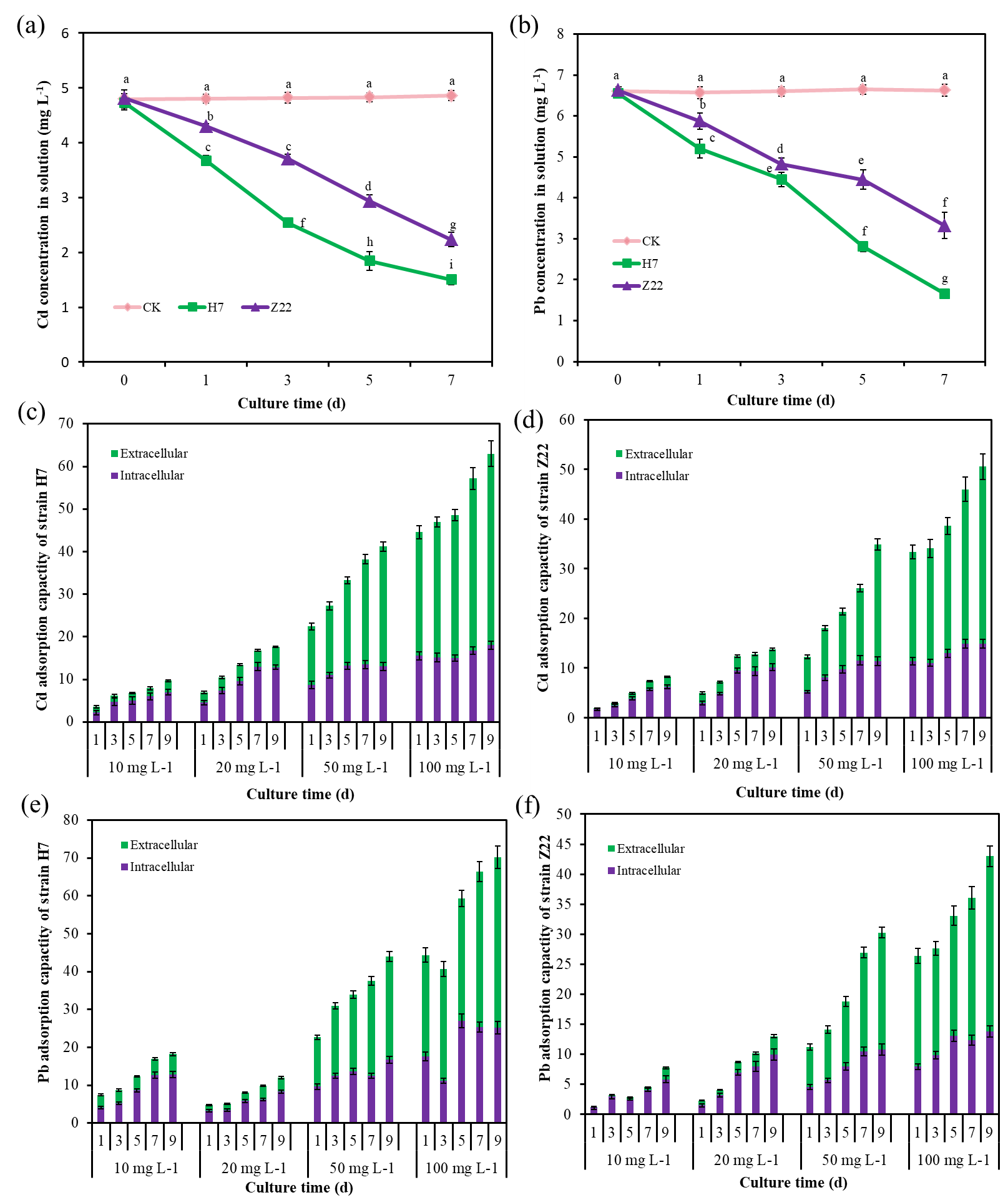


**FIGURE S3** The effects of strain H7 and Z22 on the adsorption of heavy metals. (a) The effect of strains H7 and Z22 on Cd concentration; (b) The effect of strains H7 and Z22 on Pb concentration; (c) The intracellular and extracellular content of strain H7 at different concentrations of Cd; (d) The intracellular and extracellular content of strain Z22 at different concentrations of Cd; (e) The intracellular and extracellular content of strain H7 at different concentrations of Pb; (f) The intracellular and extracellular content of strain Z22 at different concentrations of Pb. The values are presented as the means and standard deviations (n = 3). Different lowercase letters indicate statistically significant differences (*P*<0.05).


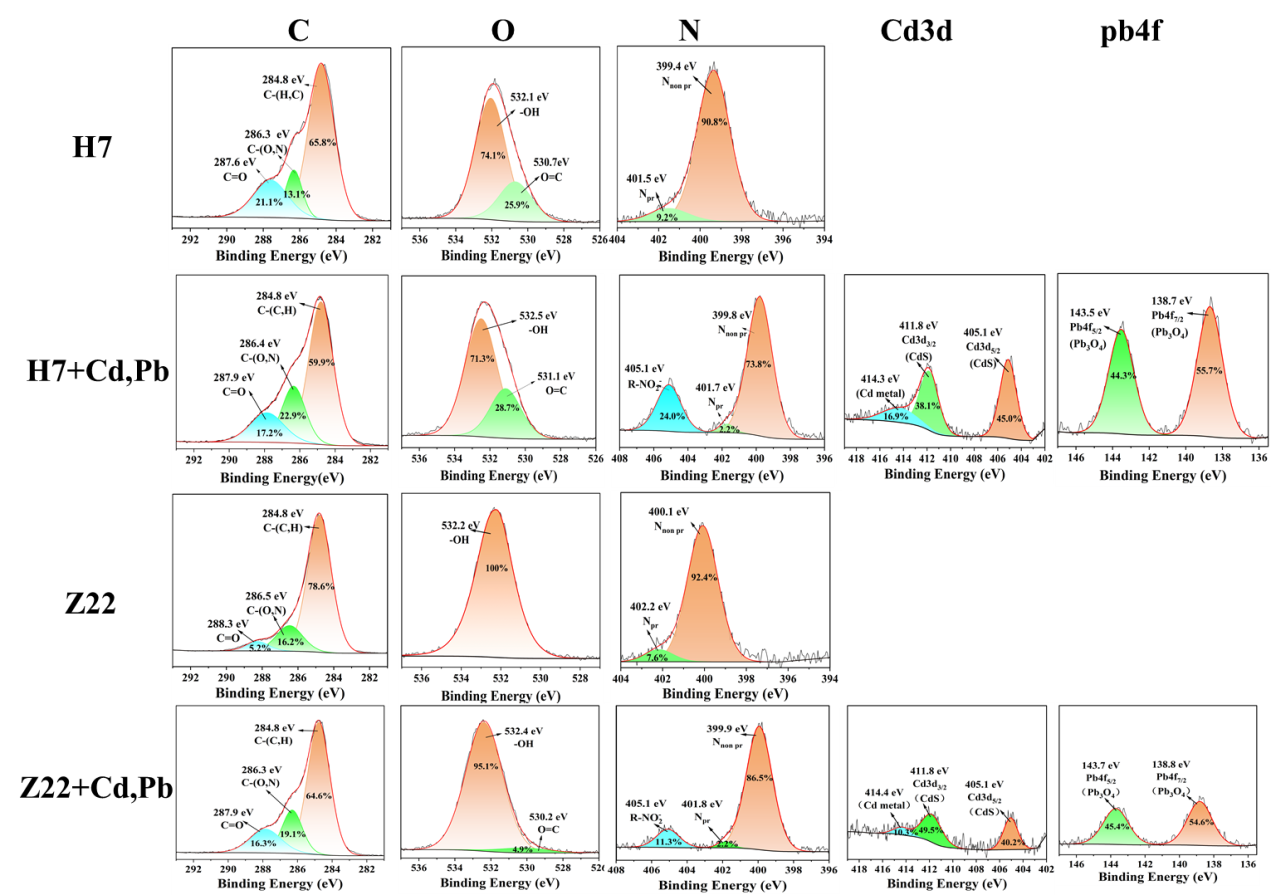


**FIGURE S4** XPS analysis for C, O, N, Cd3d and Pb4f. The red curve represents the total curve of the fitted peak, which is the superposition result of all sub-peaks. It is generated after fitting through a mathematical model (such as the Gauss-Lorentz function) and is used to analyze the contributions of different chemical states of elements and verify the accuracy of the peak fitting. The black curve represents the background baseline, which is used to separate the true photoelectron signal in the original data from the background noise generated by inelastic scattered electrons. First, adjust the background range in the Avantage software. Then, calibrate the entire spectrum based on the C1s peak binding energy. According to the chemical state binding energy of the target elements (such as C, O, N, Cd and Pb), select the Gauss-Lorentz function for peak division, and calculate the proportion of the area of each sub-peak to the total peak area. Then import the data into the Origin software to draw a graph, and the chemical peaks shown by each substance can be obtained.


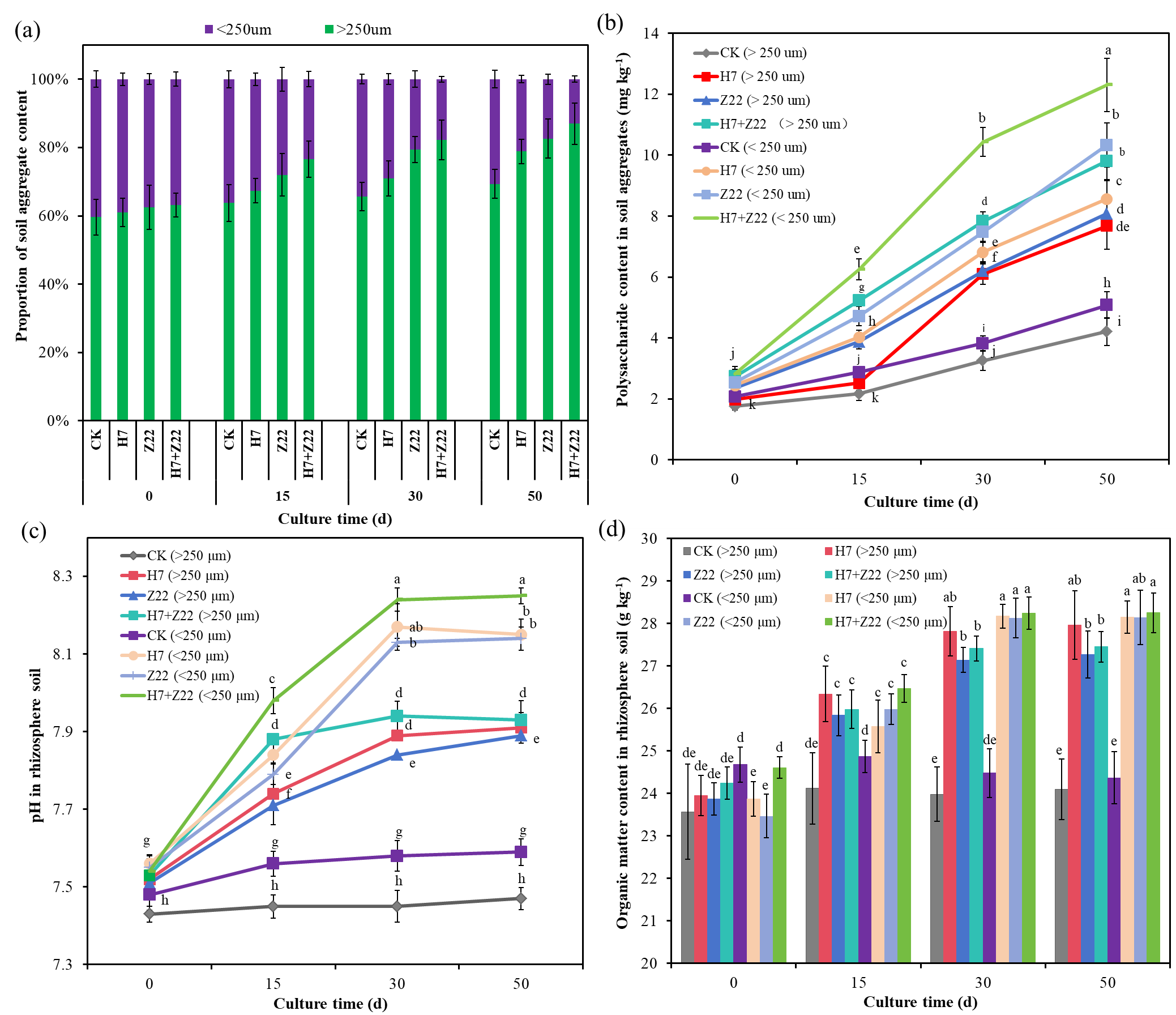


**FIGURE S5** Polysaccharide-producing bacteria on the particle size distribution and polysaccharide content of soil aggregates. (a) Particle size distribution of soil aggregates with two different particle sizes; (b) Polysaccharide content of soil aggregates with two different particle sizes. (c) pH; (d) Organic matter content. The values are presented as the means and standard deviations (n = 3). Different lowercase letters indicate statistically significant differences (*P*<0.05).


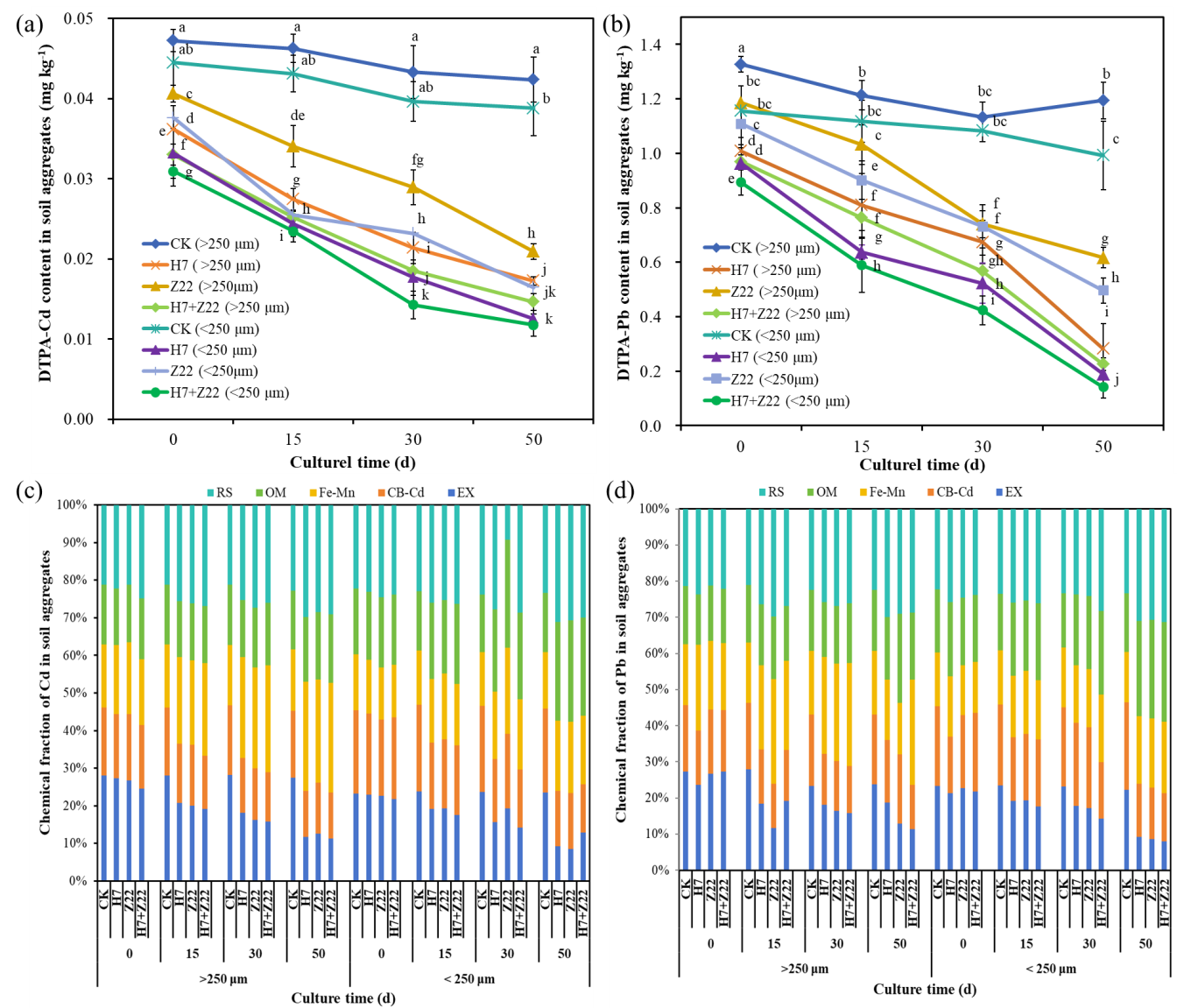


**FIGURE S6** The effect of polysaccharide-producing bacteria on the content of different forms of Cd and Pb in soil aggregates. (a) The DTPA-Cd content in soil aggregates; (b) The DTPA-Pb content in soil aggregates; (c) The distribution of different forms of Cd in soil aggregates; (d) The distribution of different forms of Pb in soil aggregates. EX-Cd/Pb: exchangeable Cd/Pb (EX-Cd/Pb); CB-Cd/Pb: carbonate-bound Cd/Pb; Fe-Mn-Cd/Pb: Fe-Mn oxide-bound Cd/Pb; OMB-Cd/Pb: organic matter-bound Cd/Pb; RES-Cd/Pb: residual Cd/Pb. The values are presented as the means and standard deviations (n = 3). Different lowercase letters indicate statistically significant differences (*P*<0.05).


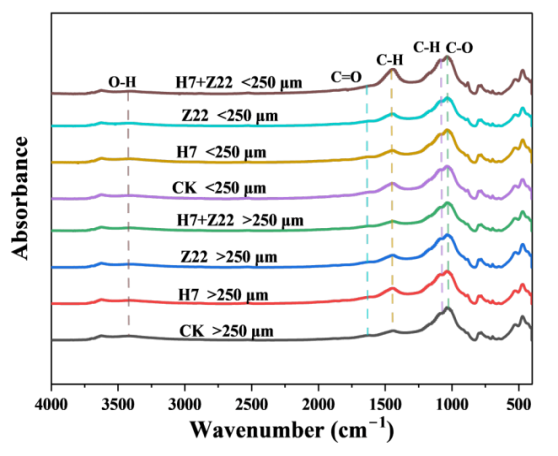


**FIGURE S7** FITR images of polysaccharide-producing bacteria immobilized Cd and Pb in soil aggregates.
